# Designing and generating a single-chain fragment variable (scFv) antibody against IL2Rα (CD25): An *in silico* and *in vitro* study

**DOI:** 10.22038/ijbms.2021.51709.11728

**Published:** 2021-03

**Authors:** Parnian Navabi, Mohamad Reza Ganjalikhany, Sepideh Jafari, Moein Dehbashi, Mazdak Ganjalikhani-Hakemi

**Affiliations:** 1 Department of Immunology, Faculty of Medicine, Isfahan University of Medical Sciences, Isfahan, Iran; 2 Department of Cell and Molecular Biology, Faculty of Biological Science and Technology, University of Isfahan, Isfahan, Iran.; 3 Division of Genetics, Department of Cell and Molecular Biology and Microbiology, Faculty of Biological Sciences and Technologies, University of Isfahan, Isfahan, Iran

**Keywords:** CD25, Daclizumab, IL-2Rα, Protein engineering, Single-chain variable - fragment (scFv)

## Abstract

**Objective(s):**

IL-2Rα plays a critical role in maintaining immune function. However, expression and secretion of CD25 in various malignant disorders and autoimmune diseases are now well established. Thus, CD25 is considered an important target candidate for antibody-based therapy. This study aimed to find the most suitable linker peptide to construct a functional anti-CD25 single-chain fragment variable (scFv) by bioinformatics studies and its production in a bacterial expression system.

**Materials and Methods:**

Here, the 3D structures of the scFvs with different linkers were predicted and molecular dynamics simulation was performed to compare their structures and dynamics. Then, interactions between five models of scFv and human CD25 were calculated via molecular docking. According to MD and docking results, the anti-CD25 scFvs with (Gly4Ser)3 linker were constructed and cloned into pET-22b(+). Then, recombinant plasmids were transformed into *Escherichia coli* Bl21 (DE3) for expression using IPTG and lactose as inducers. Anti-CD25 scFv was purified from the periplasm and detected by SDS-PAGE and Western blot. Afterward, functionality was evaluated using ELISA.

**Results:**

*In silico* analysis showed that the model containing (Gly4Ser)3 as a linker has more stability compared with other linkers. The results of SDS-PAGE, Western blot, and ELISA confirmed the accuracy of anti-CD25 scFv production and its ability to bind to the human CD25.

**Conclusion:**

Conclusively, our work provides a theoretical and experimental basis for production of an anti-CD25 scFv, which may be applied for various malignant disorders and autoimmune diseases.

## Introduction

CD25 (IL-2Rα) is a component of the heterotrimeric IL-2 receptor complex that is expressed on the surface of normal immune cells, such as regulatory T cells (Tregs), activated cytotoxic T cells, and activated Natural killer cells (NKs). In contrast, there is strong evidence for the CD25 expression and secretion in many malignant disorders and autoimmune diseases such as human-T cell lymphotropic virus-1(HTLV-1), B cell chronic lymphoblastic leukemia (CLL), acute lymphoblastic leukemia (ALL), hairy cell leukemia (HCL), Hodgkin lymphoma (HL), non-Hodgkin lymphoma (NHL), lung adenocarcinoma, head and neck cancers, as well as multiple sclerosis (MS), rheumatoid arthritis, and systemic lupus erythematosus. It is assumed that interactions of IL-2Rα with IL-2 may stimulate the growth and proliferation of tumor cells and promote tumor aggressiveness (1). Daclizumab, a humanized anti-CD25 IgG1 monoclonal antibody (mAb), has been produced for blocking this receptor and thus, inhibits the activation of the IL-2 signaling pathway (2). Although mAbs have been proven to be essential effective reagents in basic science, diagnosis, and therapy of diseases, there are many reasons for their failure when using them as therapeutic agents, such as poor penetrating abilities in solid tumors, prolonged maintenance in circulation, and difficulties in mass production (3-5). Obviously, these characteristics limit the use of mAbs as therapeutic tools. In recent years, genetically engineered antibody technology has led to advances in recombinant antibody and antibody fragment production, which are essential for research, diagnosis, and therapeutic approaches (6, 7). One of the popular forms among the engineered antibodies is single-chain variable fragment (scFv) generated by the linking of a variable heavy (VH) and a variable light chains (VL) of an antibody via a short polypeptide linker (8). Compared with the defects of mAbs, scFv has several advantages including lower molecular weight, good penetration in tissues, rapid blood circulation, and low retention in the kidney (9). Successful construction of scFv relies on the length, sequence, and flexibility of the linker peptide that could significantly affect not only the spatial structures and stability of the scFv but also the expressing level and antigen-binding activities of the scFv molecule (10-12). Therefore, choosing a functional linker among a wide range of linkers is one of the most challenging factors for generation of an optimal scFv. *In silico* studies (e.g., using molecular dynamics simulation and molecular docking) play critical roles in understanding structural-functional relationships in proteins. Of essential aspects of molecular simulation, prediction, analysis, and comparison of the 3D structure and dynamics of antigen-binding sites of antibodies could be mentioned (13, 14). 

The main purpose of this study was to compare the impact of five flexible linkers with different lengths and different degrees of flexibility on the structures and functions of single-chain antibodies and to select a suitable linker to connect the Daclizumab variable domains by computational methods. In addition, we aimed to express the scFv containing an appropriate linker in a bacterial expression system.

## Materials and Methods

***In silico methods***

*Selection of the linkers*

Peptide sequences of GSTSGSGKPGSGEGSTKG (L1), KESGSVSSEQLAQFRSLD (L2), EGKSSGSGSESKST (L3), (GGGGS)3 (L4), and GSAGSAAGSGEF (L5) with different characteristics and lengths were adopted as the linkers of the single-chain antibody in the present study (8, 15-17).

*Preparation of structures*

**Figure 1:**
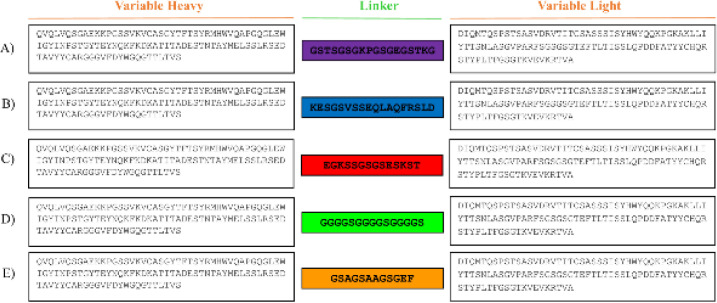
Amino acid sequence of five different scFv structures. A) scFv-L1, B) scFv-L2, C) scFv-L3, D) scFv-L4, E) scFv-L5

The crystal structure of Daclizumab (Anti IL-2Rα) was obtained from Protein Data Bank (PDB ID: 3NFP) (18). Then, the variable domains of light and heavy chains of Daclizumab were connected by adding five linkers in the structures (Figure 1). Prediction of 3D structures for five modeled scFv was done by the Robetta server (http://robetta.bakerlab.org) (19). Five probable optimal structures for each protein sequence were predicted and then the best of which was selected for further analysis. Each structure has been evaluated for stereochemical quality and residue-by-residue geometry using PROCHECK. Afterward, structural investigations were performed by Swiss-PDB viewer 4.10 and Chimera1.12 (20, 21). VMD 1.9.1 was used to visualize the trajectories and analyzing scFvs’ motions during MD simulations (22). 

*Molecular dynamics simulations*

Molecular dynamics simulations on five modeled structures and native model were performed to evaluate the dynamics and flexibility of scFv using AMBER 14 with ff14SB force field (23, 24). The structures were solvated in a truncated octahedron box with a TIP3P water model in a 10 Å hydration layer. The structures were neutralized by addition of Na^+^ ions and the coordination and topology files were saved for the next steps of MD simulations. Minimization of the system was performed in two steps. First, water and ion, then the whole system were minimized with a total of 5000 steps including 2500 steps of steepest descent, followed by 2500 steps of a conjugate gradient. Non-bonded interactions were calculated at a cutoff distance of 10 Å by PME (Particle Mesh Ewald) method in periodic boundary conditions (25). The system was heated from 0 to 300 K for 300 ps, using Langevin thermostat in NVT ensemble (26). The equilibration was performed in two steps, for 100 and then 400 ps in NVT and NPT ensemble, respectively. Finally, production MDs were done in 100 ns for five forms and the native one with the NPT ensemble. The SHAKE algorithm was used to constrain the bonds involved in hydrogen atoms. The coordinates were then saved every 20 ps.

*Analysis of trajectories*

In order to analyze the MD productions, cpptraj from AMBER Tools 15 was used (24). The root means square deviation (RMSD), fluctuation (RMSF), accessible surface area (ASA), and radius of gyration (RadGyr) were calculated for all models regarding the initial structure. 

*Molecular docking prediction*

Molecular docking prediction was performed using the Prism online tool to determine binding free energy and the residues involved in the interaction between human CD25 and anti-CD25 scFv (27). 

***Experimental methods***

*scFv fragment design*

The anti-CD25 scFv expression cassette was designed in a VH-(Gly4Ser)3-VL format plus an N-terminal periplasmic leader sequence (PelB). Two restriction sites for NdeI and XhoI enzymes were designed at its two ends. Afterward, this sequence was ordered to be synthesized by General Biosystems, USA on the base of the pET-22b(+) vector (Novagen, Inc, USA). Restriction enzyme digestion and DNA sequencing were done to ensure the presence of the insert and to verify the correct frame reading format of the recombinant construction. 

*Transformation of the anti-CD25 scFv*

The recombinant plasmids were transformed into *E. coli* Bl21 (DE3) competent cells (Novagen, USA) by the heat shock method, and the colonies were plated onto Luria–Bertani (LB) agar medium (Sigma, Co, USA) containing 100 μg/ml ampicillin. Confirmation of the transformants clones was done by the Colony PCR method. Transformed bacteria with pET-22b(+) without recombinant scFv gene were used as a mock control.

*Colony PCR*

After transformation of *E. coli* BL21 by pET-22b(+)-scFv, positive clones were selected by colony PCR. Colony PCR was carried out using specific primers. The sequences of forward and reverse primers were 5′-GGCTACACCGAGTACAACCA-3′ and 5′-GTAGTAGGTGGC GAAGTCGT-3′, respectively. Each reaction mixture (20 μl) was composed of 1 µl of culture as a template, 10 µl of 2X Taq^Basic^ PCR Master Mix2 (BioFACT, Co, South Korea), 1 µl of primers and the amplification program consisted of an initial denaturation step at 95 °C for 2 min, followed by 30 cycles of denaturation step at 95 °C for 20 sec, an annealing step at 61 °C for 40 sec, an extension step at 72 °C for 1 min, followed by a final extension at 72 °С for 5 min. Finally, the PCR product including the ScFv gene (483 bp) was shown on a 1% (w/v) agarose gel.

*Expression of the anti-CD25 scFv*

For expression of the protein, *E. coli* Bl21(DE3) harboring pET22b(+)-scFv were grown overnight in LB medium with 100 µg/ml ampicillin at 37 °C with shaking at 180 rpm. The scFv production was induced by adding isopropyl β-D-thiogalactopyranoside (IPTG; Sigma, Co, USA) (1 mM) and Lactose (5 mM) to the medium when the optical density (OD) at 600 nm reached 0.6-0.8, and growth was continued at 37 °C with shaking at 180 rpm for 2–16 hr.

*Extraction of the periplasmic Proteins*

The proteins were extracted using cold osmotic shock from periplasmic space with the following procedure: the cells were harvested by centrifugation at 7000 rcf and 4 °C for 10 min. Pelleted cells were resuspended in ice-cold TES buffer (20 mM Tris-HCl [pH 8.0], 1 mM EDTA [pH 8.0], 20% sucrose) with a volume equal to 2% of the initial volume of bacterial culture medium. After a 30 min incubation on ice, a volume equal to 3% of the initial volume of bacterial culture medium, ice-cold TES buffer which was diluted 1/5 with PBS buffer were added to previous solution and incubated on ice for 30 min. Afterward, ice-cold MgSO_4_ (50 mM) was added to the solution and after 20 min incubation on ice, the cells were centrifuged at 11000 g and 4 °C for 15 min. Supernatants were then collected and analyzed for the presence of scFv by sodium dodecyl sulfate-polyacrylamide gel electrophoresis (SDS-PAGE).

*Purification of the anti-CD25 scFv*

For purification of 6xHis-tagged scFv from cell culture supernatants, Ni-NTA affinity chromatography (Thermo Fisher Scientific, Co, USA) was used. Briefly, the Ni-NTA resin was washed with bounding buffer (20 mM Tris, 0.2 M NaCl, 10 mM Imidazole [pH 8.0]) until the pH of the effluent reached 8.0. Afterward, supernatants containing scFv were passed through the column and the proteins without 6His-tag were eluted from the column with wash buffer (20 mM Tris, 20 mM Imidazole [pH 8.0]). The scFv was finally purified using elution buffer (20 mM Tris, 0.6 M Imidazole [pH 8.0]) and proteins were then analyzed by 12% SDS-PAGE and Western blot analyses. 

*SDS-PAGE and Western blot analysis*

Anti-CD25 scFv was assessed by sodium dodecyl sulfate-polyacrylamide gel electrophoresis (SDS-PAGE) so that proteins were denatured at 95 °C for 7 min and 15 µl of each sample was loaded on 12% SDS-PAGE gel at 90 V. Then, gels were stained with Coomassie brilliant blue and silver nitrate. For Western blot, after the proteins had been separated by 12% SDS-PAGE, the proteins were transferred to a polyvinylidene difluoride (PVDF) membrane by a semidry procedure at 300 mA for 15 min and then, blocked by 5% (w/v) skimmed milk overnight at 4 °C. The membrane was rinsed 3 times with PBS-T (0.05% Tween in PBS) and incubation was performed with 1/1000 dilution of mouse anti-His-tag HRP mAb (Sigma, Co, USA) at RT for 1.5 hr. Finally, after 3 washes with PBS-T, protein bands were visualized using enhanced chemiluminescence (ECL). 

*Enzyme-linked immunosorbent assay (ELISA)*

Antigen binding analysis of scFv against CD25 was characterized using ELISA. To do this, CD25 antigen (8 μg/ml, 100 μl/well; R&D systems biotechnology, USA) in 50 mM carbonate/bicarbonate buffer, pH 9.5 was coated in a 96-well plate at 4 °C overnight, and then blocked with 2% BSA at 37 °C for 1 hr (three uncoated wells were performed as negative control). Subsequently, the scFv as the test group and mouse anti-human CD25 mAb (Daclizumab; R&D Systems Biotechnology, USA) (1;1000 dilution) as positive control were added to antigen-coated wells and incubated at 37 °C for 1 hr. After 3 washes with PBS, the reactivity of scFv was detected by adding anti-His-tag HRP antibody (1:1000 dilution; Sigma). The reactivity of positive control was clarified by anti-mouse-IgG HRP (1:1000 dilution; Sigma). The enzymatic reaction was developed with TMB and stopped at a defined time with H_2_SO_4 _(2 M) and the absorbance was measured at the wavelength of 450 nm by a microplate reader (ELX 800). 

*Inhibition ELISA*

Inhibition ELISA was performed for further confirmation of the specificity of scFv. The 96-well plate was precoated with CD25 antigen (8 μg/ml, 100 μl/well) in 50 mM carbonate/bicarbonate buffer, pH 9.5, and incubated at 4 °C overnight. Following blocking (2% BSA at 37 °C for 1 hr) and washing steps, the scFv was added and incubated at 37 °C for 1 hr. Next, the mouse anti-human CD25 antibody was added to the reaction wells and incubated at 37 °C for 1 hr. After 3 washes with PBS, anti-Mouse-IgG HRP (1:1000 dilution) was applied for detection and finally, the OD value at a wavelength of 450 nm was obtained.

*Measurement of anti-CD25 scFv affinity*

Affinity of the purified ScFv was determined using the ELISA method. Briefly, 1.5 µl of a concentration of 8 µg/ml from CD25 antigen (R&D systems, USA) was prepared in a carbonate-bicarbonate buffer (50 mM, pH 9.6). Then, each well of a 96-well microplate (Nunc, Co., Denmark) was coated by adding 100 µl of the CD25 antigen in a series of three doubling dilutions in rows A (1 = 8 µg/ml), B (½ = 4 µg/ml), and C (¼ = 2 µg/ml). After washing and blocking, the ScFv was prepared at an initial concentration of 50 µg/ml and added in a series of three doubling dilutions in columns 1, 2, and 3, respectively. HRP-conjugated anti-His antibody (Sigma, Germany) was added, and the immune reactivity was assessed with TMB substrate. The reaction was stopped with a stop solution and OD was measured at 450 nm using a microplate reader (Hyperion, Inc., USA). Finally, the affinity was calculated based on the Beaty equation (28).

***Statistical analysis***

In this study, version 20 of SPSS was used for statistical analysis. A one-way ANOVA test was applied for comparisons between samples, negative controls, and positive controls. Data are presented as the mean±SD of 3 identical repeats. *P*-values equal to or less than 0.05 were considered as statistical significance. 

## Results

***Validation of predicted model consistency with functional results***

**Figure 2:**
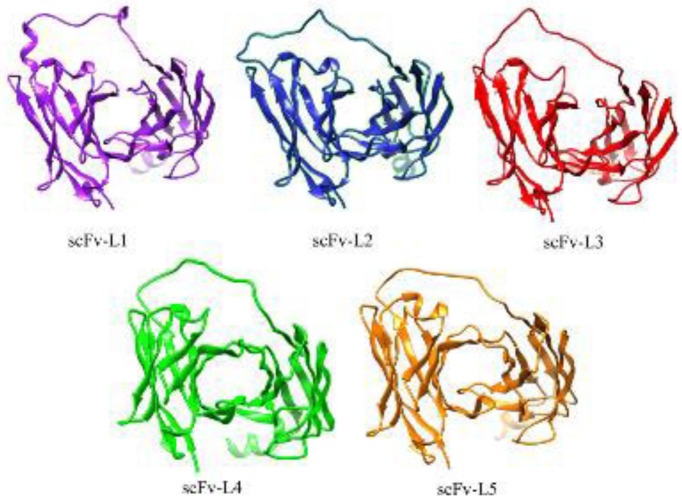
3D structures of anti-CD25 scFv with five different linkers

In order to validate the modeled scFv structures, structural superposition has been made with the native Daclizumab to verify the overall conformation and appropriate structural arrangement of light and heavy chains. Then the modeled structures have been evaluated using PROCHECK (Table supplement1-5). The 3D modeled structures of anti-CD25 scFv are shown in Figure 2.

***Analysis of molecular dynamic simulation trajectories***

**Figure 3:**
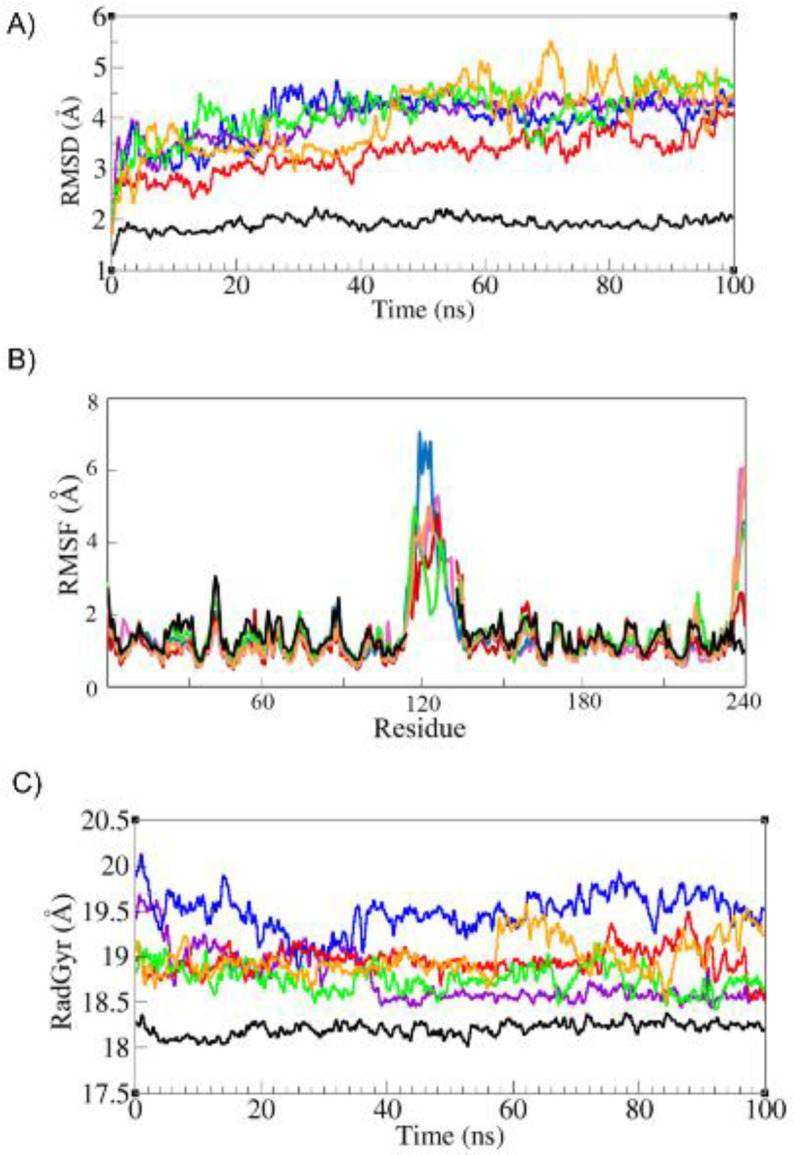
A) Root Mean Square Deviation (RMSD) of αC of all models: scFv-L1 is depicted in violet, scFv-L2 in blue, scFv-L3 in red, scFv-L4 in green, scFv-L5 in orange, and the native is shown in black. B) Root mean square fluctuation (RMSF) in all models: scFv-L1 is depicted in violet, scFv-L2 in blue, scFv-L3 in red, scFv-L4 in green, scFv-L5 in orange, and the native is shown in black. C) Radius of gyration of all models: scFv-L1 is depicted in violet, scFv-L2 in blue, scFv-L3 in red, scFv-L4 in green, scFv-L5 in orange, and the native is shown in black

**Figure 4:**
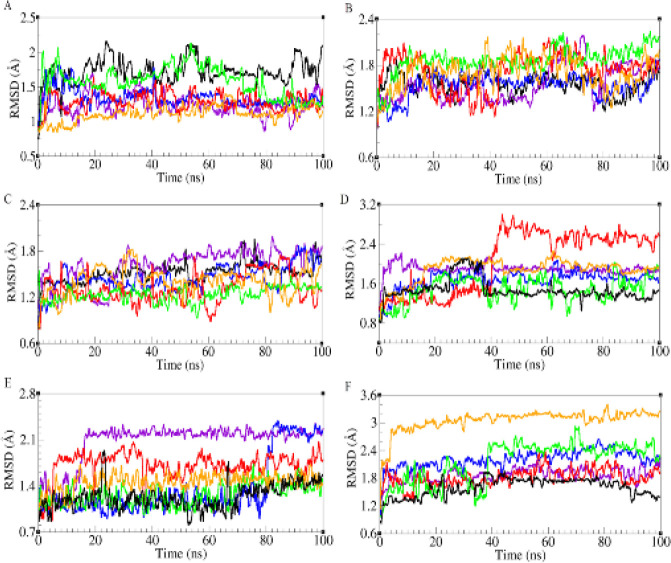
RMSD of CDRs in all models. scFv-L1 is depicted in violet, scFv-L2 in blue, scFv-L3 in red, scFv-L4 in green, scFv-L5 in orange, and the native is shown in black. VH-CDR1 (A), VH-CDR2 (B), VH-CDR3 (C), VL-CDR1 (D), VL-CDR2 (E), and VL-CDR3 (F)

RMSD: root means square deviation; CDRs: complementarity determining regions

**Figure 5:**
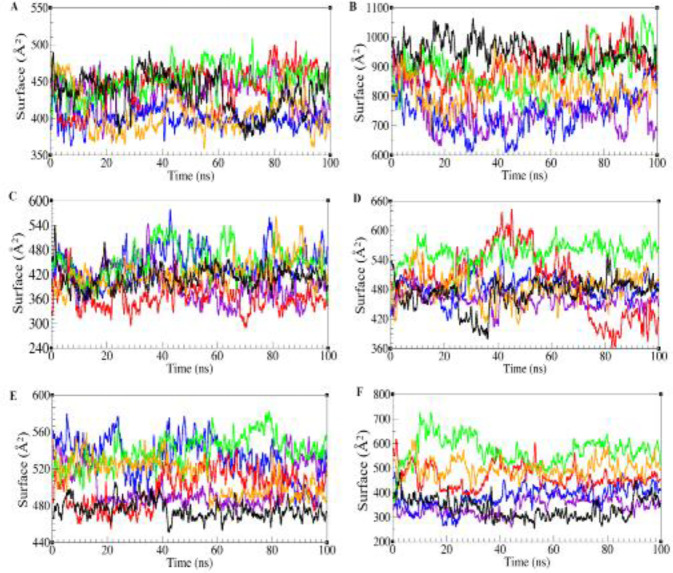
Accessible surface area of CDRs. scFv-L1 is depicted in violet, scFv-L2 in blue, scFv-L3 in red, scFv-L4 in green, scFv-L5 in orange, and the native is shown in black. VH-CDR1 (A), VH-CDR2 (B), VH-CDR3 (C), VL-CDR1 (D), VL-CDR2 (E), VL-CDR3 (F)

After selection of best predicted models, the trajectories of all five models and native structure (Daclizumab) were analyzed using RMSD (root mean square deviation), RMSF (RMS fluctuations), ASA (accessible surface area), and RadGyr (radius of gyration). RMSD calculation was used to measure the stability of the structures during the simulation. The backbone RMSDs of the whole structure and complementarity determining regions (CDRs) of all structures have been depicted in Figure 3A and Figure 4. As depicted in Figure 3A, the RMSD value for native structure remains stable around 2 Å during the simulation and the RMSD values varied between 2.5 to 5 Å for five modeled scFvs. The RMSD of scFv-L3 stays around 3 Å during the simulation with the lowest fluctuations regarding other scFv structures and the RMSD for scFv-L5 shows the highest fluctuations in comparison with other structures. Also, scFv-L1, scFv-L2, and scFv-L4 show moderate RMSD values during the simulation. Complementarity-determining regions (CDRs) are immunoglobulin (Ig) hypervariable domains in variable chains (VL/VH), where these molecules have the most extensive interactions with their specific antigens (29). The CDR regions of VH were located at positions 27-35, 48-68, and 93-110, and the CDR regions of VL were located at positions 24-33, 47-55, and 88-96 (30). RMSD values of CDRs in both chains were depicted in Figure 4. The RMSD values for CDR1 and CDR2 of VH in native structure and five models were between 1 to 2.5 Å during the simulation. Also, the RMSDs values of VH-CDR3 were between 1 to 1.8 Å for all models and scFv-L4 showed the least RMSD value during the simulation. RMSD values of VL-CDR1 were between 1 to 2.5 Å, but the scFv-L3 RMSD value increased sharply at 40 ns and then remained around 3 Å. The RMSD values of VL-CDR2 in scFv-L1 and scFv-L3 showed more fluctuations. The RMSD value for scFv-L4 has the same value for the native structure. Also, in VL-CDR3, scFv-L5 showed a larger conformational movement than other linkers. RMSF values of all models were assessed in order to investigate the contribution of linkers in the dynamical behavior of the overall structures (Figure 3B). scFv-L2 had the most flexibility which fluctuated up to 7 Å compared with other structures. scFv-L4 had the lowest flexibility regarding other structures. Since CDRs in both chains have the most binding contributions to the antigen, the accessible surface area study (ASA) was carried out. As shown, the accessible surface areas of CDR1, CDR2, CDR3 in both chains are depicted in Figure 5. ASA values of VH-CDR1 for scFv-L2 and scFv-L5 decreased over time more than other models. The lowest ASA values belong to VH-CDR2 of scFv-L1 and scFv-L2. Also, the ASA value for VH-CDR3 of scFv-L3 was decreased during the simulation. The ASA values for VL-CDR1 of scFv-L3 and scFv-L4, were increased from the beginning of the simulation until the end. VL-CDR2 of scFv-L2 and scFv-L4 had the highest ASA values. The ASA values of VL-CDR3 of scFv-L1 and scFv-L2 were decreased, while the highest ASA value belongs to L4.

To describe the compactness of all structures, the radius of gyration (Rgyr) was measured (Figure 3C). Rgyr values ranged from 18 to 20 Å for each structure during simulation and scFv-L4 had constant value over the simulation time, which depicts similar behavior as the native structure.

The average structure from the final 100 frames of the simulation from each construct has been extracted and aligned with daclizumab (Figure S1 supplementary material).

***Molecular docking analysis***

Molecular docking of five models of anti-CD25 scFv was performed and the differences in their binding modes were investigated. The involved residues in the interaction and binding free energies between anti-CD25 scFv and human CD25 were predicted. The lowest binding free energy (higher affinity) was observed for anti-CD25 scFv-L5 (-69.96 energy score) and scFv-L4 (-67.33 energy score) as compared with other models. On the contrary, the highest binding free energy was observed for anti-CD25 scFv-L2 (-52.82 energy score).

***Construction and Verification of pET-22b (+)-scFv***

**Figure 6:**
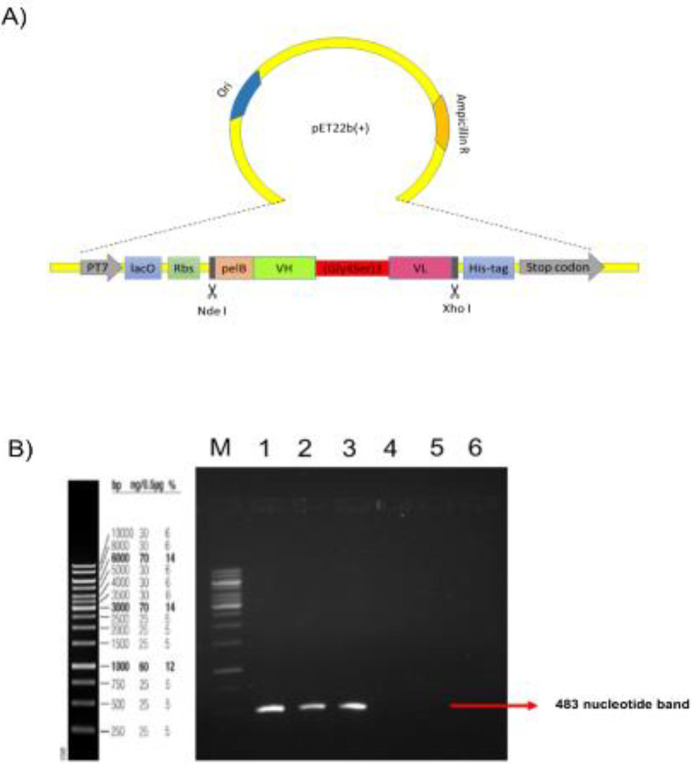
A) Schematic representation of recombinant scFv Plasmid: The scFv gene (VH-(Gly4Ser)3-VL) was inserted into the pET22b(+) vector. The positions of T7 lac promoter (PT7), ribosome binding site (Rbs), restriction enzyme sites, pelB leader (pelB), 6-histidine tag (His-tag), and stop codon are illustrated. B) Colony PCR amplification of scFv gene: PCR products were electrophoresed in a 1% w/v agarose gel and visualized with safe stain (Invitrogen, USA).Lane M: DNA marker (GeneRuler SM342-500); Lanes 1-3: Transformed colonies with plasmid pET-22b(+) with recombinant scFv gene; Lanes 4-6: Transformed colonies with plasmid pET-22b(+) without recombinant scFv gene (mock control). The 483 bp compared with controls supports the presence of recombinant scFvin transformed colonies

To construct the anti-CD25 scFv, the amino acid sequences were derived from the variable light and variable heavy chains of the Daclizumab monoclonal antibody and were joined by (Gly4Ser)3 linker. The constructs contained the pelB leader sequence at the N-thermal and the His-tag sequence at the C-terminal, thus, allowing the accumulation of expressed proteins in the periplasm and the detection or purification of the expressed protein. We selected pET-22b(+) as an expression plasmid and *E. coli* Bl21(DE3) as a host cell. The schematic diagram of pET22b(+)-scFv is shown in Figure 6A. The anti-CD25 scFv was synthesized and cloned into the pET-22b(+) vector by the General Biosystems company. The recombinant scFv plasmids were transformed into *E. coli* strain Bl21(DE3) competent cells by the heat shock method and the colonies containing plasmids with ampicillin resistance gene were screened for insertion by digestion using NdeI and XhoI restriction enzymes, electrophoretic analysis, and DNA sequencing (not shown in the figure). Hence, we confirmed that pET-22b(+)-scFv has been successfully constructed.

***Determination of the presence of pET-22b (+)-scFv in bacterial colonies***

For further verification of the presence of anti-CD25 scFv in *E. coli* BL21, colony PCR was performed. As expected, the PCR products indicated the presence of the right-sized DNA insert. (Figure 5B).

***Characterization of anti-CD25 scFv***

**Figure 7:**
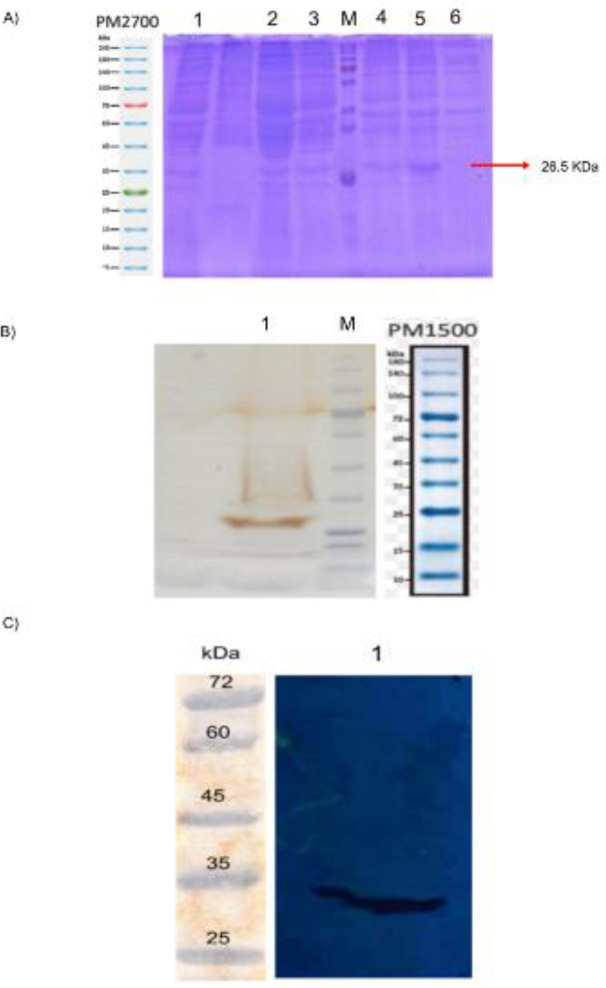
SDS-PAGE and Western Blot Analysis. (a) Coomassie brilliant blue staining of the total periplasmic proteins, Lane M: Protein size marker (SMOBIO PM 2700); Lanes 1-5: Expression of scFv protein at 2, 4, 6, 8, and 16 hr after induction with IPTG and Lactose (The anti-CD25 scFv band is marked with an arrow); Lane 6: Proteins of transformed bacteria before induction with IPTG and Lactose. (b) Silver staining of purified anti-CD25 scFv from Ni-NTA column, Lane M: Protein marker (SMOBIO PM 1500); Lane 1: purified scFv. (c) Western blot analysis was carried out with an anti-His tag antibody against the 6-His tag for detection of anti-CD25 scFv, Lane M: Protein marker (SMOBIO PM 2700); Lane 1: Purified ani-CD25 scFv was visualized using enhanced chemiluminescence (ECL)

After extraction of the periplasmic fractions and purified scFv, samples were analyzed using SDS-PAGE and Western Blot methods. The outcome of SDS-PAGE and Western blot analysis demonstrated that scFv was detected in both the periplasmic and purified proteins with a molecular weight of approximately 26.5 kDa (which was as anticipated). The results are shown in Figure 7. 

***Biological activity of anti-CD25 scFv against human CD25***

**Figure 8:**
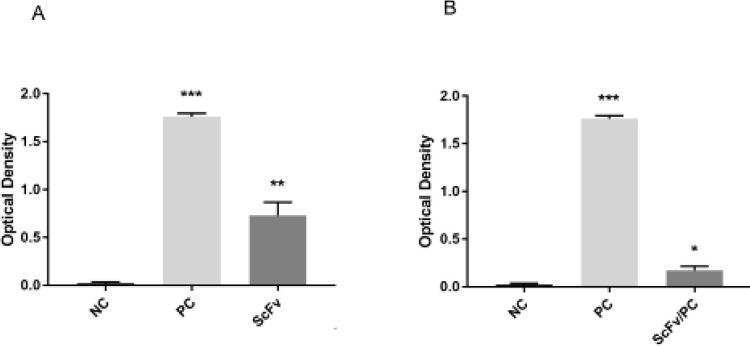
ELISA detection of anti-CD25 scFv activity. Human CD25 antigen was bound on the well bottom, and after blocking and washing, the ability of anti-CD25 scFv and Daclizumab (positive control) to bind CD25 was assessed. Un-coated wells were used as a negative control. In this experiment, One way ANOVA was performed to compare data sets using the SPSS software. (A) Direct ELISA: the binding activity of anti-CD25 scFv to CD25 antigen was significantly increased compared with the negative control (*P*=0.002). (B) Inhibition ELISA: in the presence of scFv, connection of Daclizumab to CD25 was significantly reduced (*P*=0.018)

Indirect ELISA analysis showed that anti-CD25 scFv exhibited a considerable binding activity to human CD25 antigen (*P*=0.002) (Figure 8A). The outcome of inhibition ELISA analysis showed that Daclizumab’s connection to the CD25 antigen was significantly reduced in the presence of scFv (*P*=0.018) (Figure 8B). This confirmed that scFv was bound to the same epitope reacted with Daclizumab. 

***Binding affinity of anti-CD25 scFv***

Applying the ELISA method and the Beaty equation, the binding affinity of the anti-CD25 scFv was calculated as 5.01×10^-7^ M. 

## Discussion

The scFv fragments, consisting of the variable domains of the heavy and light chains, are used for the diagnosis and treatment of malignancy, cancer research, and biomedical applications (31). The scFv molecules have a single antigen-binding site and an approximate molecular weight of 25 kDa (32). These smaller fragments exhibit several advantages such as good affinity, rapid diffusion into solid tumors, and short retention times. (33). The length and sequence of the linkers are two essential aspects that can significantly influence the correct folding, stability, and expression level of scFv (34, 35). It has also been reported that stability and affinity of scFv have a direct correlation (36). In general, it is important to investigate the effects of different linkers on the stability and activity of scFv. Therefore, one basic issue in this study was the comparison of stability and other properties of scFv structures with five different flexible linkers using computational studies and selection of the best linker for making an optimal anti-CD25 scFv, which maintains the biological activity of scFv. In this study, homology modeling and MD simulation were applied to compare the structure and stability of all anti-CD25 scFv models. The RMSD graphs of structures indicated that scFv-L3 retained overall stability throughout 100 ns of simulation and showed the least RMSD compared with other structures over time compared with the native model while scFv-L5 showed more fluctuations. Comparison of RMSD graphs of CDR regions illustrated that approximately in all CDRs, scFv-L4 was very similar to the native structure during simulation, which indicated that scFv-L4 and native structure possessed a very similar binding affinity with the human CD25 antigen. Based on the RMSF graphs, the anti-CD25 scFv-L4 construct was more stable with lower structural fluctuations in the linker region. Meanwhile, because of the structural importance of CDR for antibodies, we calculated the accessible surface area for CDRs. The result implied that scFv-L4 is better able to bind to human CD25 than other models because almost all CDRs in this construct had increased ASA values which provide a better opportunity to interact with CD25. Based on Rgyr values, it has been found that anti-CD25 scFv-L4 is stable over time and behaves very similar to the native structure. Additionally, molecular docking has been used to evaluate the binding affinity of each designed anti-CD25 scFv with human CD25 protein. Based on docking results, the interaction patterns in all five models and native structure were similar and scFv-L4, scFv-L5 were better than others. In fact, the best candidate has been chosen considering several structural and dynamical parameters simultaneously, including the overall dynamics of the constructs, the relative CDR distances, the amount of flexibility and surface accessibility of CDRs, and the binding affinity. To summarize, comparison of MD results and docking studies presented in this study suggest that anti-CD25 scFv with the L4 is more stable and has a favorable binding energy and interaction pattern. An important issue in the production and efficient expression of scFv is selecting an appropriate expression host from various expression systems. Several expression systems are available for the production of scFv, including bacteria, yeast, and mammalian cells (37-39). It should be noted that each of these hosts has advantages and disadvantages (40). Nevertheless, *E. coli* is one of the widely used hosts for the expression of antibody fragments (41). Several FDA-approved biopharmaceuticals, including Certolizumab pegol (Cimzia), Ranibizumab (Lucentis), and Abciximab (ReoPro) are produced using bacterial systems (42-44). Fast growth rates, high yield of the product, easy genetic manipulation, and cost-effectiveness of *E. coli *host have made it a reliable and precious tool as an expression system (38, 45). Previous studies have indicated that proteins expressed in the cytoplasm of *E. coli* often form insoluble inclusion bodies that do not have proper folding (46-48). This inefficiency in producing soluble scFv is caused by the inability to form disulfide bonds under the reducing conditions of *E. coli* cytoplasm (37, 48). The Intra-domain disulfide bonds of scFv play crucial roles in the correct folding and structural stability (49, 50). Therefore, there are a number of strategies that can be employed for the successful expression of efficient scFv. Secretion of proteins into the bacterial periplasm (using fusion to an N-terminal leader peptide such as pel B) which has an oxidizing environment that allows the formation of their disulfide bridges, is one strategy to increase solubility and correct folding of scFv (51-54). Several studies have demonstrated the successful expression of effective scFv within the *E. coli* host (37, 39, 55). Also, Miller *et al*. used *Saccharomyces cerevisiae*, *Pichia pastoris*, and *E. coli* to express human scFv antibodies. Their results showed that expression in *E. coli* was the fastest and the most stable way to determine structure-function relationships of scFv (39). These reasons prompted us to choose *E. coli* periplasm as the expression host in this study for the production of anti-CD25 scFv. Our SDS-PAGE and Western blot results showed that the selected positive *E. coli* clones expressed the 26.5 kDa soluble form anti-CD25 scFv (Figure 7). Furthermore, antigen-binding of scFv demonstrated that anti-CD25 scFv can specifically bind to CD25. Based on the indirect ELISA data (Figure 8A) the performance of the anti-CD25 scFv was slightly lower than that of the Daclizumab. This difference in binding strength can most probably be attributed to the monovalent nature of the binding activity of scFv while monoclonal antibodies consist of two binding sites for each antigen (56, 57). As previously reported, the affinity of different antibodies is in the range of 10^-7^ to 10^-10 ^M. Specifically, the affinity of Daclizumab has been reported as 3×10^-10^ M (58). In the current work, the Ag-Ab affinity constant (K) of anti-CD25 scFv was calculated as 5.01×10^-7^ M. Regarding the fact that ScFv, in contrast with mAb, has only one antigen-binding capacity, the binding activity of our ScFv could be considered an appropriate affinity. 

## Conclusion

All in all, computational studies appear to indicate that scFvs containing different linkers exhibit different properties, and the model containing (Gly4Ser)3 as a linker (L4) has been chosen as the best option among other linkers. Moreover, our experimental studies on the expressed anti-CD25 scFv in *E. coli* Bl21 (DE3) periplasmic space confirmed the successful production and functionality of the scFv. This scFv derived from the monoclonal antibody Daclizumab as a treatment strategy may be used to target various CD25-positive malignant disorders and autoimmune diseases.
